# Acute changes of metabolic parameters after fluid challenge

**DOI:** 10.1186/cc14258

**Published:** 2015-03-16

**Authors:** T Nguyen, D De Bels, M Pustetto, P Cottignies, J Devriendt, C Pierrakos

**Affiliations:** 1 Brugmann Hospital, Brussels, Belgium

## Introduction

The detection of heart response to fluid administration is still a challenge in clinical practice. Changes in metabolic parameters may be useful to detect changes in cardiac output (CO) after fluid expansion.

## Methods

This is a prospective observational study in adult critically ill patients. CO was measured either by echocardiography or by a thermodilution method (PiCCO, Swan-Ganz catheter). Hemodynamic measurements and blood gas analysis were obtained before and after a fluid challenge with either 1,000 ml crystalloid or 500 ml colloid. Arterial and central venous blood gas samples were taken simultaneously. Oxygen delivery (DO_2_), oxygen consumption (VO_2_) and carbon dioxide production (VCO_2_) were calculated according to well-known formulas. Patients were divided into three groups (high responders, mild responders and nonresponders) according to their change in CO (>20%, 10 to 20%, <10%, respectively).

## Results

We evaluated 27 patients, age 68 (95% CI: 61 to 74) and APACHE II score 22 (95% CI: 18 to 26). Seven patients were high responders, eight patients were moderate responders and 12 were nonresponders. DO_2_ was significantly increased in high responders (37 ± 35%, *P *< 0.01) as compared with moderate responders or nonresponders. Furthermore, nonresponders had a decrease in their DO_2_ (-10 ± 7%, *P *< 0.01), while moderate responders showed no change in their DO_2 _(1.6% ± 10, *P *= 0.73) after fluid challenge. We found no differences in changes in lactate levels and central venous oxygen saturation (ScvO_2_) between high responders, moderate responders and nonresponders. No differences in the changes of VCO_2_ or VO_2_/VCO_2_ ratio were found between high responders, mild responders and nonresponders too. Changes in DO_2_/VCO_2_ ratio were found to be significantly increased only in high responders (47 ± 73% vs. -14 ± 31%, *P *= 0.02) and not in mild responders (15 ± 54% vs. -14 ± 31%, *P *= 0.15) as compared with nonresponders.

## Conclusion

Only significant increases of CO (>20%), after fluid administration, lead to improved oxygen delivery; DO_2_ may be decreased in nonresponders. The changes of ScvO_2_ and lactate levels do not track the changes of CO after fluid challenge. The DO_2_/VCO_2_ ratio may be a useful index to identify significant increases of CO after fluid challenge in cases where CO measurement is not feasible.

